# Preparation of a reference material for microplastics in water—evaluation of homogeneity

**DOI:** 10.1007/s00216-021-03198-7

**Published:** 2021-02-06

**Authors:** John Seghers, Elzbieta A. Stefaniak, Rita La Spina, Claudia Cella, Dora Mehn, Douglas Gilliland, Andrea Held, Ulf Jacobsson, Håkan Emteborg

**Affiliations:** 1 European Commission, Joint Research Centre (JRC), 2440 Geel, Belgium; 2 European Commission, Joint Research Centre (JRC), 21027 Ispra, Italy

**Keywords:** Reference material, Microplastics, Water, Homogeneity, PET, Harmonisation

## Abstract

Validation of analytical methods for measurements of microplastics (MP) is severely hampered because of a general lack of reference materials, RM. There is a great need to develop such reference materials. This study presents a concept of three-component kit with immobilised MP in solid NaCl, a surfactant and clean water that can be applied for the production of many types of MP RMs. As proof of concept, an RM for polyethylene terephthalate (PET) particles in water was prepared and evaluated for its homogeneity. The particles ranged from 30 μm (Feret_min_) to about 200 μm adapted by wet sieving. A specific number of PET particles were immobilized in about 0.29 g of solid NaCl by freeze-drying 1 mL of a NaCl suspension. By using manual and automated counting, twenty reconstituted 1-L water samples were evaluated for homogeneity with respect to number of PET particles from 30 μm to > 200 μm/L of water. The number of particles was 730 ± 120 (mean ± one standard deviation (SD); *n* = 10) and 865 ± 155 particles (*n* = 10) obtained by optical microscopy in two independent laboratories. This corresponded to relative SDs of 16.4 and 17.9% and a mean of 797 ± 151 particles (18.9% RSD, for *n* = 20). Homogeneity studies of the NaCl carrier without reconstitution resulted in 794 ± 60 particles (7.5% RSD). The homogeneity of PET in the salt carrier was also evaluated directly with respect to mass of PET per vial using an ultra-micro balance. An average mass of 293 ± 41 μg of PET was obtained (14, % RSD for *n* = 14). Micrographs were recorded to demonstrate the absence of major sources of contamination of the RM components. Information about the particle size distribution and particle shapes was obtained by laser diffraction (LD) and dynamic image analysis (DIA). In addition, the identity of the PET polymer was confirmed by Raman and FT-IR spectroscopy. The RM was developed for a large-scale inter-laboratory comparison of PET particles in water (ILC). Based on the homogeneity results, the material was found to be sufficiently homogeneous to be of meaningful use in the ILC. In a 3-day process, more than 500 samples of PET particles in the NaCl carrier were prepared with good potential for further upscaling with respect to the number of vials or with other kinds of polymers. The stability of PET was not evaluated but it was deemed to be stable for the duration of the ILC.

**Supplementary Information:**

The online version contains supplementary material available at 10.1007/s00216-021-03198-7.

## Introduction

Contamination of the environment with microplastics (MP) has gained significant attention among the general public in the last few years. MP is mainly generated by degradation of larger plastic objects and is gradually becoming a substantial global problem [1]. Microplastic particles have been found in all compartments of the aquatic and terrestrial environments where they accumulate and may remain for a long time. There is also growing concern that MP presents a risk to the environment and human health via contamination of water, food, soil and air [2].

Microplastics represent a diverse range of material types of different shapes, colours and sizes. Consequently, MP is not a well-defined entity and there is no consensus on a definition [3, 4]. Frias and Nash proposed a definition for microplastics: “Microplastics are any synthetic solid particle or polymeric matrix, with regular or irregular shape and with size ranging from 1 μm to 5 mm, of either primary or secondary manufacturing origin, which are insoluble in water” [3]. Likewise, Hartmann et al. listed recommendations and proposed a definition and a categorization framework in order to achieve consensus [4]. Primary microplastics are originally manufactured to be of small size (for example, microbeads in cosmetics) while secondary microplastics originate from the breakdown of larger plastic items which is eventually the main source of MP [5]. Koelmans et al. and a report from the WHO describe various findings of the type and number of MP particles in different kinds of water samples which is interesting to compare with the current work with respect to the levels found [6, 7].

To manage the waste cycles of plastics and to diminish the release of MP into the environment, multiple aspects of plastics have become a major policy in many EU Member States and of the European Commission [8].

Reliable monitoring of MP contamination in relevant matrices would be necessary for efficient implementation of different policies related to microplastics. A major obstacle for obtaining comparable monitoring data is the lack of standards, (certified) reference materials and harmonised techniques that facilitate comparability of MP measurements. It is particularly challenging to prepare reference materials that are mimicking MP found in environmental samples since such MP particles are of irregular shape, fragmented, oxidised and sometimes covered with biofilms. The JRC (Joint Research Centre) of the European Commission and BAM (Bundesanstalt für Materialforschung und prüfung, DE) therefore launched an ambitious inter-laboratory comparison (ILC) for the measurement of irregular PET particles in water.

In general, in any exploratory work, it is better to start with less complex samples. Therefore, as a first step, the aim was to evaluate the potential of different measurement techniques for MP measurements in a simplified sample matrix which comprised one polymer (PET) in a specific size range and one major matrix component (water).

The test item sent to the ILC participants must be sufficiently homogeneous and stable in order to provide meaningful (or at least comparable) results [9, 10]. Due to its hydrophobic properties, MP immersed or suspended directly in water is notoriously difficult to homogenise in the absence of surfactants. To keep MP homogenously distributed while aliquoting from a large recipient would be challenging as the MP would float on the surface or agglomerate. Therefore, an approach to prepare a test item for direct analysis was not attempted (e.g., to supply of a water bottle containing MP particles). Instead, a sample kit with three different components was devised to provide the ILC participants with homogeneous (i.e., similar) test items. The three components were (i) 0.29 g of solid NaCl carrier containing a specific number/mass of PET particles, (ii) a rinsing solution containing a Triton X-100 surfactant and (iii) a glass bottle filled with 950 mL pure water. The participating laboratories had to transfer the PET particles into the water bottle by using 50 mL of the rinsing solution applying a detailed reconstitution protocol prior to immediate analysis. The final volume of the sample was therefore 1 L.

A concept previously developed by Elordui-Zapatarietxe et al. was adapted by subsampling of a vigorously stirred NaCl suspension with PET particles and a surfactant [11]. In the current study, the number and mass of the particles per litre of water were the quantities intended to be measured (measurands) as explained in the results and discussion for parameters A–F [12, 13]. Subsequent freeze-drying of the suspension immobilised the PET particles in NaCl. In this way, each sub-sample from the PET suspension was firmly retained in 10-mL vials containing a solid NaCl carrier. The kits were placed at 4 °C after arrival in the participants’ laboratories. The PET particles had then to be quantitatively transferred to the bottle of water using the surfactant rinsing solution. It was known that about 0.29 g of salt was present in each vial. Therefore, the mass of salt was also recorded by the ILC participants as a quality control check. The full results of the ILC will be reported elsewhere. Nevertheless, some important information related to the ILC will be provided as this paper also aims to illustrate the full concept and workability in preparing this kind of RMs for a larger ILC.

The current work describes the preparation of the three-component kit and the results of the homogeneity study of the resulting reference material to be used in the ILC. The homogeneity studies were performed on fully reconstituted water samples and by direct analysis of the NaCl carrier. Evaluation of the particle size distribution and particle shape descriptor of the PET is also reported. To verify the identity of the PET polymer, unambiguous spectral identification is also presented.

Existing RMs in this area are very scarce, although reference material 8634 (ethylene tetrafluoroethylene for particle size distribution and morphology) provided by NIST (Gaithersburg, MD, USA) was initially envisaged for validation of some of the measurement techniques used in this work. Unfortunately, NIST 8634 was designed for a different purpose and the particles are mainly below 30 μm. The number population and amount of sample in NIST RM 8634 is not comparable to the material developed here. Interestingly, the relative expanded uncertainties for the 25 and 30 μm size classes are 22 and 24%, respectively.

## Materials and methods

### Cryogenic milling and adaptation of particle size fraction of the PET particles

Granular polyethylene terephthalate (PET), of type Lighter C93, (standard bottle grade), was produced by Equipolymers (Schkopau, DE) and provided by BAM (Berlin, DE) through IK Industrievereinigung Kunststoffverpackungen e.V. (DE).

To produce microplastic particles of a desired size range from about 30 to 200 μm, a combination of cryogenic milling and different sieving steps were necessary. To this end, 20 g of PET granulate was cryo-milled using a SPEX 6875 Freezer/Mill (Metuchen, NJ, USA). The PET granulate was ground through 45 cycles of 3 min with a cooling period of 5 min in between each step. After milling, the PET powder was transferred into a glass beaker with a glass cover to prevent contamination by airborne particles.

A wet-sieving method was then used to operationally define and separate the 30–200 μm PET particle size fraction from smaller and larger particles. This approach was used since wet sieving allows electrostatically charged and agglomerated particles to be more efficiently separated. To achieve this, 2 g of cryogenic-milled polyethylene terephthalate (PET) was suspended in 500 mL water + 1 mL Triton X-100 and stirred for 2 h with an overhead mixer and stainless steel propeller. The batch was then wet-sieved over a stack of sieves (180 μm and 45 μm, respectively), and the particles retained on the 45-μm sieve mesh were subsequently washed with type 1 water (as defined by ASTM [14]) to remove excess surfactant. Type 1 water must also pass a final filter of 0.22 μm to fulfil this requirement.

To further reduce the number of particles < 30 μm, and to remove any remaining particles > 200 μm, a second wet-sieving step was performed by re-suspending the particles retained on the 45-μm sieve mesh in 500 mL water + 1 mL Triton X-100 and stirring for 2 h. The suspension was then wet-sieved again over a stack of sieves (180 μm and 45 μm, respectively). The particles retained on the 45-μm sieve were rinsed again with type 1 water. The remaining particles were finally re-suspended in 1 L of type 1 water with 0.5 mL of Triton X-100 surfactant to make up the stock suspension of PET particles for the ILC. Measurements with a QICPIC system (Sympatec, Clausthal-Zellerfeld, DE) based on dynamic image analysis showed about 2000 particles > 30 μm per mL of suspension. A fraction of small MP particles < 30 μm were still present. Nevertheless, the PET material was still considered suitable for its use in the ILC.

### Freeze-drying of the suspension with PET MP

The resulting suspension with PET was further diluted with type 1 water up to 1 L to obtain a suspension containing 800 particles/mL. Next, NaCl (31434-M, Ph. Eur., ≥ 99.5%, Sigma-Aldrich, BE) was added to obtain a 29.5% (m/v) salt solution. Mixing was performed using an overhead stirrer with a stainless steel propeller (to avoid milling effects observed with glass-coated magnetic stirring bars). By keeping the suspension of particles under vigorous mixing with a 0.1% (m/m) Triton X-100 surfactant in the NaCl solution, repeatable subsampling of PET particles was possible since the hydrophobic particles were homogeneously distributed in the bulk solution. Aliquots of 1 mL of the suspension were transferred to 10-mL amber glass vials which had been cleaned with a Miele Neodisher detergent and rinsed with type 1 water. After washing, the vials were dried in a clean cell to avoid contamination with airborne particles and/or fibers (Terra Universal, Fullerton, CA, USA). Likewise, PTFE-coated rubber inserts were washed with Miele Neodisher detergent, rinsed with type 1 water, and dried in the clean cell.

The vials were filled in a clean bench using a 1000 μL pro-pipet with glass tip. In total, 521 vials were filled with 1 mL aliquots of the saturated NaCl/PET suspension. The vials were then placed in a Martin Christ Epsilon 2-10D freeze dryer (Osterode, DE). A 3-day drying program was applied to convert the PET-containing salt solution into a dry cake (NaCl carrier) of about 0.29 g salt. After freeze-drying, the vials were sealed with the PTFE-coated inserts and secured with aluminium caps. (The choice of PTFE-coated inserts may seem contradictory, but it is harder than rubber and provides an acceptable seal, and since the inserts are fixed, they do not contaminate the salt cake with small pieces of PTFE). The vials were labelled with unique identification numbers according to fill order and samples were taken randomly over the whole filling sequence for checking of homogeneity of PET in the material before and after reconstitution.

### Bottles with water and vials with Triton X-100 surfactant solution

The bottles and PTFE-coated screwcaps were cleaned and dried as described above for the vials. The 1-L borosilicate glass bottles were filled with 950 ± 1 mL type 2 water (as defined by ASTM [14]). Type 2 water was chosen as more than 500 L was required to fill all water bottles and the production capacity of type 1 water was insufficient. The filled volume was controlled by mass using a custom-made filling system (Teblick, Antwerp, BE). The inside of the cylindrical 550-L container used in this system is covered with perfluoroalkoxy polymer (PFA). A CMP StarKleen™ in-line filter with a removal efficiency of particles down to 5 μm was placed just before the water entered the glass bottle (Pall, Port Washington, NY, USA). The filling itself took place in a clean bench from Nuaire (NU-164, Plymouth, MN, USA).

A 0.1% (v/v) Triton X-100 Merck (VWR, BE) solution was filled in pre-cleaned 100-mL vials (cleaned and dried as given above). A 100-L stainless steel container was cleaned and rinsed with diluted Triton X-100 solution and type 2 water. Thereafter, 35 L of 0.1% Triton X-100 solution was prepared directly in the cylindrical vessel. The solution was gently stirred during 2 h with a stainless steel propeller before transfer to a 20-L glass bottle. Using a dispenser connected to the 20-L glass bottle, 60 mL portions of the Triton X-100 solution was filled in the 100-mL vials.

### Reconstitution protocol to obtain test item with PET for the ILC

The participants had to verify that all solid content was located at the bottom of the small vial with the NaCl carrier. If this was not the case, the vial had to be shaken/tapped to transfer the solids to the bottom. Thereafter, the vial had to be opened and made sure that the insert was intact. One portion of 5 mL of Triton X-100 solution was then used to rinse the inner side of the insert of the small vial directly into the 1-L glass bottle. This was done to transfer any particles that were attached to the insert.

Thereafter, one portion of 5 mL of Triton X-100 solution was added to the vial. The vial was rotated and gently shaken to dissolve the salt carrier and disperse the PET content, while avoiding the formation of foam. The resulting suspension was poured into the 1-L glass bottle by rotating the vial to ensure quantitative transfer of the contents. This step was repeated nine times, so the total volume of Triton X-100 solution used was 50 mL.

### Optical microscopy for counting of particles

At JRC-Geel, seven NaCl carrier samples were analysed by dissolving the salt with the 0.1% Triton X solution directly in the vial. The suspension holding the particles was transferred directly onto a 47-mm 0.2-μm Whatman black polycarbonate track edge filter (VWR, BE). The vial was rinsed ten times with 5-mL portions of the Triton X solution and three times with 5 mL type 1 water to ensure quantitative transfer of all the PET particles. One out of the ten 5-mL portions of Triton X solution was used to rinse the inside of the PTFE-coated insert and transferred any PET particle(s) present onto the filter. The filter funnel was additionally rinsed with type 1 water to capture all PET particles on the filter membrane.

The PET particles retained by the filter membrane were counted using dark field microscopy. Photos were taken with a × 6 magnification through a Zeiss stereo microscope, STEMI 2000-C (Munich, DE). In this this way, particles < 30 μm were hardly visible but matching with a certified length scale was also performed. About 15 images were manually stitched together to make up the whole filter area. The stitched images per filter were printed on A3 paper after which the particles were counted manually.

At JRC-Ispra, the ILC reconstitution protocol described above was followed. In order to minimise contamination, all steps of sample reconstitution and filtration were performed in a laminar flow hood, located inside a clean room. Glassware was cleaned with Hellmanex® III detergent (purchased from Sigma-Aldrich), then rinsed with ultrapure water (type 1 water, Millipore, filtered 0.2 μm). No plastic devices were used to reconstitute the samples and filter them. After reconstitution, the suspension of PET particles in the 1-L glass bottle was partially transferred to a 250-mL glass cylinder and then poured through the filtering system (Millipore Glass Microanalysis Filter Holder). The PET suspensions were filtered over 25 mm Whatman Nuclepore Track-Etch membranes (cat. No. 110659, Sigma-Aldrich). These steps were repeated until the whole volume of the PET suspension had been filtered. To ensure quantitative transfer of all the PET particles, the glass bottle was filled with an additional 190 mL of pure water and 10 mL of 0.1% v/v Triton X-100. The solution was used for rinsing the bottle and the glass cylinder and was then filtered as well. The remaining 10 mL solution of Triton X-100 was finally used to wash the funnel of the filtering system using a glass pipette. The polycarbonate filter was transferred to a glass petri dish and immediately analysed by dark field microscopy (Imager M2, Zeiss), objective 5×. Two images of each filter were obtained with a coupled CCD camera and recorded with 1 × 1 and 4 × 4 binning, respectively. Each full picture was composed of 1600 tiles, stuck together by the dedicated function implemented on the software. Images were then analysed by counting particles using a Java-based image processing program (ImageJ). Data analysis was carried out independently by two operators using two approaches resulting in four data sets per vial from JRC-Ispra.

1. Manual counting of the particles, performed on the 1 × 1 binning image, with the “Multi point” ImageJ function.
2. Semi-automatic counting, performed on the 4 × 4 binning image, by using the ImageJ program function “analyse particle”.

Firstly, each image was converted from RGB to 8-bit image, and the calibration factor from micron per pixel was established by measuring the length of the scale bar reported in each picture. A selection was drawn on the image to exclude the scale bar and the external part of the filter from analysis. Each operator then followed personalized procedures to optimize particle detection. Operator 1: brightness/contrast was manually adjusted until particles were visible as a white shadow on a black background; then, the threshold was set manually to cover each individual particle on the picture. Operator 2: the “auto” function on the dedicated brightness/contrast menu was selected, and the MaxEntropy threshold on dark background was applied. A so-called Watershed function was selected by both operators to separate particle images from sticking to each other. If considered suitable, the fill holes function (under the menu “binary”) was also added. Finally, the “analyze particle” function was applied with the settings: size 1000-infinity, circularity 0.0 to 1.0, and the Feret’s diameter options shown in the Results. Outlines were checked manually before accepting them and analysis was eventually repeated with small modifications of the procedure when background interfered with particle detection. The total particle number was obtained by eliminating results for Feret_min_ < 30 μm. Unfortunately, it was not possible to record a macro to completely automatize the measurement.

At an independent external laboratory, the reconstitution protocol devised for the ILC was also followed as given above. Filtration of the reconstituted water sample was carried out with a vacuum filtration system and a Buchner type funnel. A polycarbonate (PC) membrane (47 mm, Nuclepore^TM^ Whatman, 0.4 μm) was positioned at the bottom of the funnel to recover particles in suspension from the reconstituted water sample. The filter was then removed from the funnel and placed in a sterile Petri dish on the microscopic glass slide. After filtration of each sample of reconstituted water, the membranes were stored in a closed Petri dish in a desiccator at 15 °C. The filters were prepared maximum 24 h before analysis. The filters were then analysed by optical microscopy.

The recognition of particles using optical microscopy was done in transmission mode. The magnification and contrast adjustment for counting of the particles was set to observe particles > 30 μm. The transparency of the membrane was sufficient to provide a contrast which allowed distinguishing the particles as dark silhouettes on the membrane. An Olympus BX41microscope was coupled with a colour video camera to record optical images with a × 10 magnification. The × 10 objective allows visualization of objects > 25 μm in diameter and provided a lateral resolution of 8 μm which was sufficient to analyse 30-μm-sized particles. The diameter of the membrane (47 mm) was too large to record one single extended video image by the software (LabSpec 6—Horiba). Therefore, two extended video images were recorded, each corresponding to one-half of the membrane.

Although the particles were clearly distinguishable from the substrate, the contrast was not high enough to run in automated mode using the Particle Finder software (Horiba). Instead, for particle recognition and counting, the selection of particles > 30 μm was made by manual observation. The magnification of the images corresponded to areas of 5000 by 2800 μm along the X and Y axes, respectively. The entire half filter was scanned and the analysed area was verified using the X and Y coordinates for each movement. A total number of 124 zones were scanned for the whole filter. The error on the particle number was estimated from one part of the tested samples (1/4 of the filter) by comparing the counting result obtained at × 10 with a × 20 magnification. The error in the particle number was estimated to be around 4%; hence, the semi-automated mode used in this methodology provided the number of particles > 30 μm. The size of each single particle (to obtain a particle size distribution) was not measured because as explained above, the contrast of the optical images was not sufficient to run the image analysis software automatically.

### Preparation of macroporous silica membranes for weighing of PET

Macroporous silica membrane filters (1 × 1 cm, pore size of 5 μm with a pore inter-distance of 12 μm and 500 μm thickness) were obtained from Smart Membranes, Halle, DE. The empty filters were weighed as supplied using the scheme described in the next section. For filtering of the PET suspensions from the NaCl carrier, each filter was placed in a filter holder resting on a glass frit with sealing rings on each side (one made of PTFE and the other one of rubber). The filter holder was coupled to vacuum pump trough a vacuum flask. Thereafter, a glass recipient of 20 mL volume was attached with a clamp over the sealing rings and filter. The NaCl carrier with PET was dissolved using 50 mL of the 0.1% Triton X-100 surfactant and rinsed thoroughly several times to transfer all particles to the filter. All filtering took place in a Nuaire NU-164 clean bench. Thereafter, the filters were dried at 60 °C overnight in a Petri dish with a slightly open lid using an oven (Carbolite, LHT5/120, Hope, UK).

### Substitution weighing of MP-PET with an ultra-micro balance

Weighing was performed on an ultra-micro balance UMX5-model (Mettler-Toledo AG, Greifensee, CH) using the principle of substitution weighing as described in OIML R111 [15]. This balance has a maximum load of 5 g with 0.1 μg resolution. Firstly, the empty filters were weighed and later the same filters loaded with PET particles were weighed again. Weighing of the filters was performed as a comparison weighing with a combination of working mass standards of OIML class E2 (internal calibration certificate E3886) to give 90 or 92 mg (close to the empty mass of the filters, numbers rounded here for readability). Each weighing cycle per filter took about 10 min and the mass of the reference weights was recorded four times and the mass of the filters with or without PET was also recorded four times (2 × ABBA as described in OIML R111, annex C). Weighing of the filters was always bracketed by weighing of reference weights at the beginning and at the end of each weighing cycle. After each cycle, four values were obtained for the empty and loaded filters, respectively, by calculating the difference to the reference weights. Thereafter, the masses of the empty filters were subtracted from the loaded filters. The resulting masses corresponded (mainly) to PET present in the NaCl carrier. Air pressure, relative humidity and temperature were monitored and recorded. The expanded uncertainty of each weighing was evaluated as given in ISO/IEC GUIDE 98-3:2008 using GUM workbench taking the following uncertainty components into account: uncertainty of mass standards, dispersion from the weighing process, uncertainty from the balance and uncertainty in air parameters [16, 17].

### Identification of PET particles by FT-IR and Raman microscopy

A Bruker Hyperion FT-IR microscope (Milano, IT) equipped with single-element detector and a 15× IR objective was applied to collect spectral information about the PET particles. Particles deposited by filtration on a 25-mm Anodisc filter (pore size 0.1 μm) were analysed in transmission mode by subtracting the filter spectrum as background at 64 scans. Spectra were baseline subtracted, truncated at 1320 and 4000 cm^−1^using the OPUS 8.5 software, exported and compared to a spectral database [18] using the freeware, siMPle [19], in the wavelength ranges of 1320–1900 and 2700–3300.

A WITec alpha 300 Raman microscope (Ulm, DE) equipped with a 532-nm laser and 10× objective was used to collect Raman spectra of the PET particles deposited on an Anodisc filter. Ten spectra collected at 1-s exposure time were averaged, baseline subtracted and compared to Raman spectra of a home-built polymer library by using the ACD/UV IR manager software.

### Micrographs of macroporous silica membranes

Four photos of the macroporous silica membranes were taken with a × 6 magnification with the Zeiss stereo microscope, STEMI 2000-C. One additional photo was taken with a × 40 magnification to provide a close-up of the PET particles.

### Evaluation of particle size distributions, particle shape and particle number in the selected size interval by instrumental analysis

Apart from microscopy, three additional analytical techniques were used to evaluate the particle size distribution, particle shape and the particle number. Laser diffraction (LD) was performed using a Helos KR system from Sympatec (Clausthal-Zellerfeld, DE). To obtain sufficient signal intensity, 18 vials NaCl carrier with PET were dissolved in 50 mL 0.1% Triton X-100 contained in a 50-mL measurement cuvette. Vigorous stirring at 1200 rpm and brief ultrasonication was necessary to dissolve all salt. Equivalent sphere diameters (ESD) of PET particles from 0.5 to 875 μm were measured by using lenses R3 and R5 as the particles swirl around in the measurement cell describing equivalent spheres. Both the volume distribution and the number distribution were evaluated using the Sympatec Windox software. The Fraunhofer equation was applied, so no correction for refractive index was necessary.

A particle counter from PAMAS SVSS (Rutesheim, DE) was used to count PET particles in dissolved NaCl carrier using the Triton X-100 surfactant solution. The PAMAS particle counter is operating based on light obscuration had been calibrated with latex spheres in the 1–400 μm range. For these measurements, 15 vials with NaCl carrier were dissolved in 70 mL of 0.1% Triton X-100 and the sample was passed through the particle counter.

The combined surfactant solutions from the 18 vials from LD and leftovers of the 15 vials as described above were finally measured by dynamic image analysis (DIA) using a QICPIC system with a LIXCELL flow-through cuvette (Sympatec, DE). A configuration with the M5 measurement range meant that particles from 1.8 to 2000 μm could be measured with the LIXCELL flow-through cuvette where shape information can be obtained from 3.4 μm upwards. At the low particle size end, the particle imaging is limited by the pixels of the camera. This technique records an image of every particle in solution by using high frequency video images of the silhouettes from the PET particles when re-circulating the suspension through the measurement cuvette. After recording, many different particle selection criteria can be applied using the PAXOS software. As a result, particle shape, aspect ratios and particle size distribution were obtained and evaluated using the Sympatec PAXOS software.

## Results and discussion

### Considerations for the reference material preparation

Cryogenic milling is necessary to grind elastic and rubbery materials and/or temperature-sensitive materials like polymers. In cryogenic milling systems, the materials are continuously cooled well below their glass transition temperature and made brittle with liquid nitrogen. At low temperature, the material is then pulverized by mechanical impact. (The glass transition temperature of PET is 73–78 °C that will easily be exceeded in a regular impact mill operated at room temperature.)

### Considerations for measurement parameters to be reported for the microplastic reference material

It is important to realise what is being measured when applying different techniques and it is an important consideration for the developed concept. The participants of the ILC were therefore asked to report up to six different parameters denoted A–F (depending on the applied technique). Where A was the number of PET particles range > 30 μm, B was the number of all plastic particles of the specified size and C was the total number of particles > 30 μm. Similarly, for parameter D, the participants were asked to report the mass of PET; for parameter E, the mass of plastic particles; and for parameter F, the total mass of particles in the test item. A balance can strictly speaking only record parameter F, but efforts were made during the preparation of the components in the kit to only introduce PET particles. The preparation was taking place in clean benches and filtration of the components was performed so that the solid content in the NaCl carrier essentially only corresponded to PET. Consequently, the RM was designed so that parameter A was almost identical to parameters B and C. Likewise, parameter D was almost identical to parameters E and F, respectively. The numerical ranking of results would logically be C ≥ B ≥ A and F ≥ E ≥ D. This work describes the *number of PET particles* in 1 L of water and *mass of PET* in 1 L of water since contribution from other solid material in the samples was insignificant.

### Evaluation of homogeneity and indicative ranges

**Fig. 1:**
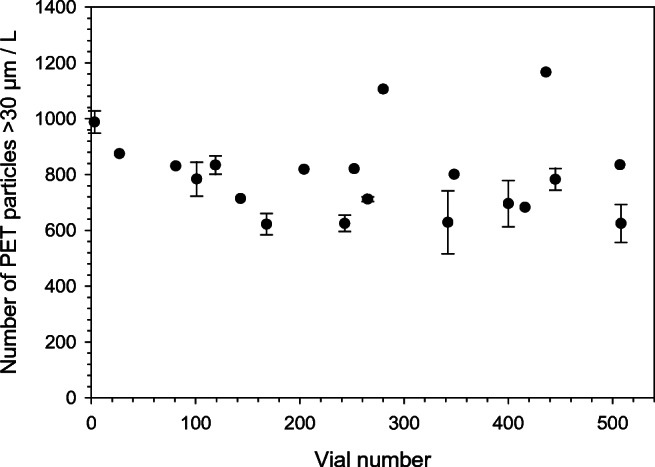
Counting results of PET particles by optical microscopy from 1-L water sample after full reconstitution of NaCl carrier, data with error bars ± 1 SD from JRC-Ispra (*n* = 4, same sample was evaluated by two different operators using two different approaches). The other data points were obtained from an external independent laboratory where one evaluation per water sample was made

**Fig. 2:**
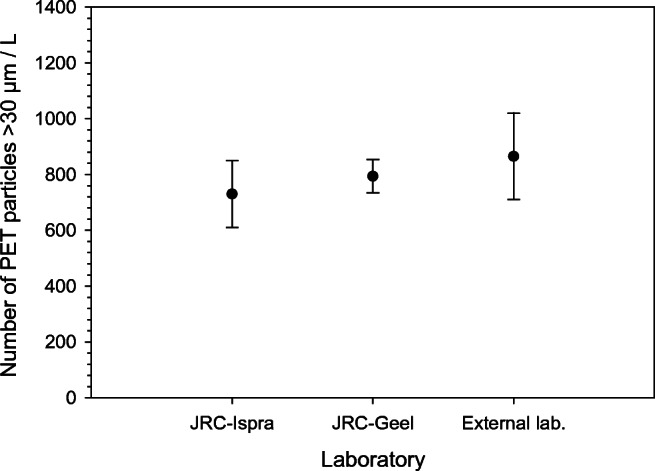
Combined counting results of PET particles in reconstituted water samples and direct analysis of NaCl carrier. JRC-Ispra and external laboratory performed a full reconstitution for *n* = 10 each. For JRC-Geel, *n* = 7 direct filtering of NaCl carrier followed by counting. All data reported with error bars of ± 1 SD

In total, 41 samples out of 521 NaCl carrier with PET were checked by manual and automated particle counting and by recording the mass (27 + 14 samples, respectively). This means that about 7.5% of the total number of samples were evaluated with respect to homogeneity. ISO 17034 indicates that the cubic root or at least ten units of the total number of samples in a reference material batch should be checked [20]. Evaluation of both between and within unit heterogeneity is desirable when checking homogeneity, which can be obtained depending on the design of the homogeneity study. In this case, no subsampling was possible since the whole bottle should be analysed as one replicate Therefore, a regular set up using analysis of variance (ANOVA) could not be performed [21]. Instead, the high number of units investigated partially compensated for this. An estimate of the homogeneity for the number of particles was 19% RSD (one SD) from the 20 fully reconstituted water samples as given in Table 1. In addition, for the mass recording, an RSD of 14% for fourteen replicates was obtained as also shown in Table 1. Abundant information was therefore available for this RM preparation before it was sent out to the participants. This reduced the risk that a specific sample would be affected by a gross error. Consequently, the prepared batch was sufficiently homogenous despite the challenging task of preparing a reference material for MP in water samples. Figure 1 illustrates all counting data for the 20 reconstituted samples as a function of vial number in the filling sequence. Results from two independent laboratories are displayed. JRC-Ispra evaluated the same filter four times, which made it possible to obtain an average and standard deviation of those observations. The 1 × 1 binning resulted in higher resolution images that helped the operator in the manual selection of the particles. On the contrary, this kind of resolution hindered the automatic analysis with the ImageJ software because of particle shape and surface roughness. With the highest resolution, particle surfaces presented the highest number of shaded areas. When applying the threshold setting, this resulted in many apparent ‘holes’ in the particles so the software could not depict each particle correctly. When using the 4 × 4 binning, particle surfaces were more uniform in colour and the automatic analysis was much improved. The data points with error bars of ± 1 SD correspond to data obtained from JRC-Ispra. From visual inspection of Fig. 1 and Fig. 2, no obvious trends exist in the data towards higher vial numbers. Significance tests of the slopes for both number and mass were also calculated using SoftCRM (a custom-made software for evaluation of homogeneity, stability and characterisation data) [22]. Before the statistical test, it was verified that the input data was normally distributed. The slopes as a function of vial number were not statistically different from zero at *P* = 0.05 using a Grubbs test for the number and mass of particles in the water samples. SoftCRM output for homogeneity (S_bb_, which in this set up was identical to the standard deviation) was 14 and 19% for the mass and number of particles as already mentioned.

**Table 1:** Indicative ranges from − 2SD to + 2SD from averages for number of PET particles > 30 μm (*n* = 20) and mass of PET (*n* = 14) per L of water. Raw data for the number and mass is reported in Table S1 and Table S2, respectively (see ESM)

Parameter	Average	SD	RSD, %	Indicative range^1^
Number/L	797	151	19	500–1100
Mass/L	293 μg	41 μg	14	210–370 μg

^1^The max and min values of the indicative ranges were rounded slightly for simplicity

To provide indicative ranges of the number and mass of particles in the reference material, the averages ± 2 standard deviations as obtained from the homogeneity studies were communicated to the ILC participants. The ranges are given in Table 1. It should be noted that the ranges by no means correspond to certified ranges with rigorously evaluated uncertainties. Based on results from numerous experiments using different techniques, the ranges are nevertheless reasonable estimates of the number and mass of PET particles in this reference material. By applying ± 2SD, one accounts for 95% of the observations. Even so, one result out of twenty for particle counting fell outside the indicative range as can be seen in bold in Table S1 (see Supplementary Information (ESM)). The indicative range for counting is wider than for weighing, which is not surprising, since the counting measurements included the reconstitution step encompassing several additional experimental steps. For the weighing data, no result was outside the indicative range which is testimony to the higher precision for those measurements as can also be seen in Table S2. Figure 2 also points in this direction, and results obtained for samples without reconstitution were measured with higher precision as obtained by JRC-Geel. A between unit heterogeneity of 19% for particle counting was obtained for test items that had undergone full reconstitution into 1 L of water. Direct analysis of the NaCl carrier without reconstitution resulted in 7.5% between unit heterogeneity for the particle counting. Additional data supporting the results for counting was obtained using the PAMAS particle counter. In this case, 870 particles from 30 to 250 μm were found which is in good agreement with the other data, although this single value is not shown in Fig. 2. That data was also collected by direct analysis of the dissolved NaCl carrier and did not encompass a full reconstitution into 950 mL of water.

### Expanded uncertainty of weighing and stability of PET

**Fig. 3:**
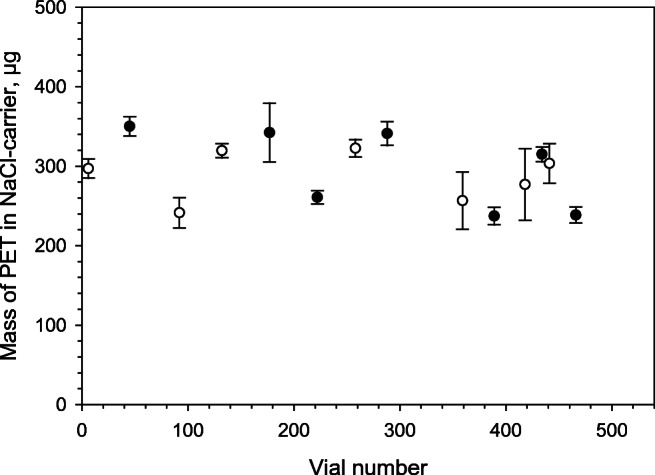
Mass of PET on macroporous silica membranes after dissolution and filtration of NaCl carrier. Error bars are ± expanded uncertainty of about 12 μg or 4% in relative terms per substitution weighing. Some error bars have larger uncertainties due to experimental difficulties when performing the weighing under too dry conditions. Filled circles: data from January, open circles data from October

The expanded uncertainty was about 12 μg for a weighing of about 300 μg of PET as can be seen from the error bars in Fig. 3. On average, each weighing was associated with a relative expanded uncertainty of 4%, (*k* = 2). Occasionally, larger uncertainties were encountered which were related to losses of PET particles due to weighing manipulations. The macroporous silica membranes had to be moved between weighing pan and a Petri dish four times for every weighing cycle as already described. The risk for inadvertent losses of material increased substantially at a relative humidity (RH) below 30%.

In this study, seven mass recordings of freshly prepared filters took place in January 2020 and a second batch of seven recordings of already prepared filters took place in October 2020. No significant difference between these results was observed as the difference between the mean values for the different periods was only 10 μg, whereas the expanded uncertainty was 12 μg per weighing. This provides indirect evidence that the PET particles were stable for 9 months at room temperature. Figure 3 shows all weighing data as a function of vial number in the filling sequence. Filled circles show weighing data from January 2020, and open circles show data from October 2020. No trend in the data was observed as evaluated using SoftCRM which also confirms that it is possible to prepare more than 500 samples from the same suspension.

### Assessment of contamination of the type 2 water and surfactant solution

**Fig. 4:**
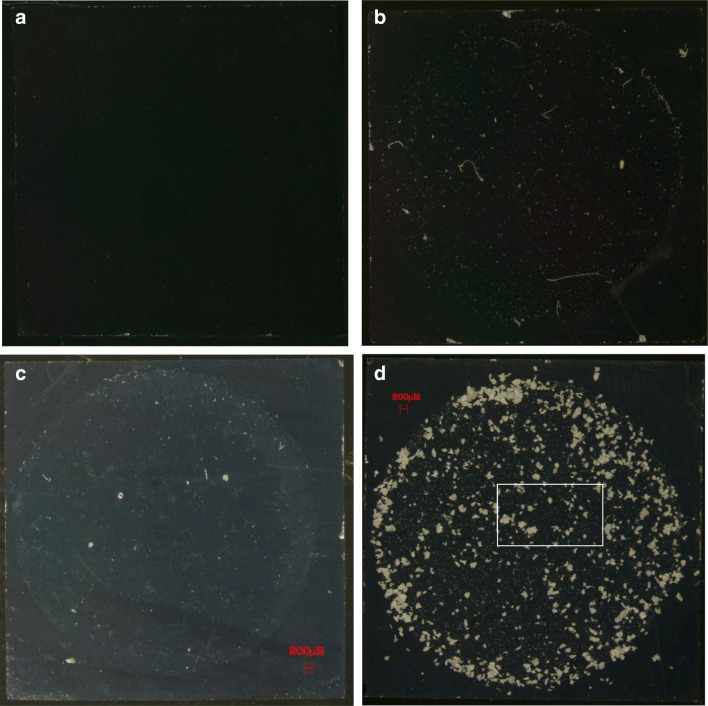
**a**–**d** All micrographs **a**–**d** were taken with a × 6 magnification. The black squares are basically only displaying the 1 × 1 cm silica membranes. **a** Micrograph of empty macroporous silica membrane before any filtering, **b** filter after filtering of 50 mL of 0.1% Triton X-100 (unit 368), **c** filter after 950 mL type 2 water (unit 369) and **d** filter after 0.29 g dissolved NaCl carrier with PET unit 369 + 50 mL of Triton X-100 (unit 369). Images **b**–**d** above with kit number in parenthesis. White rectangle in **d** is the magnified area in Fig. 5

**Fig. 5:**
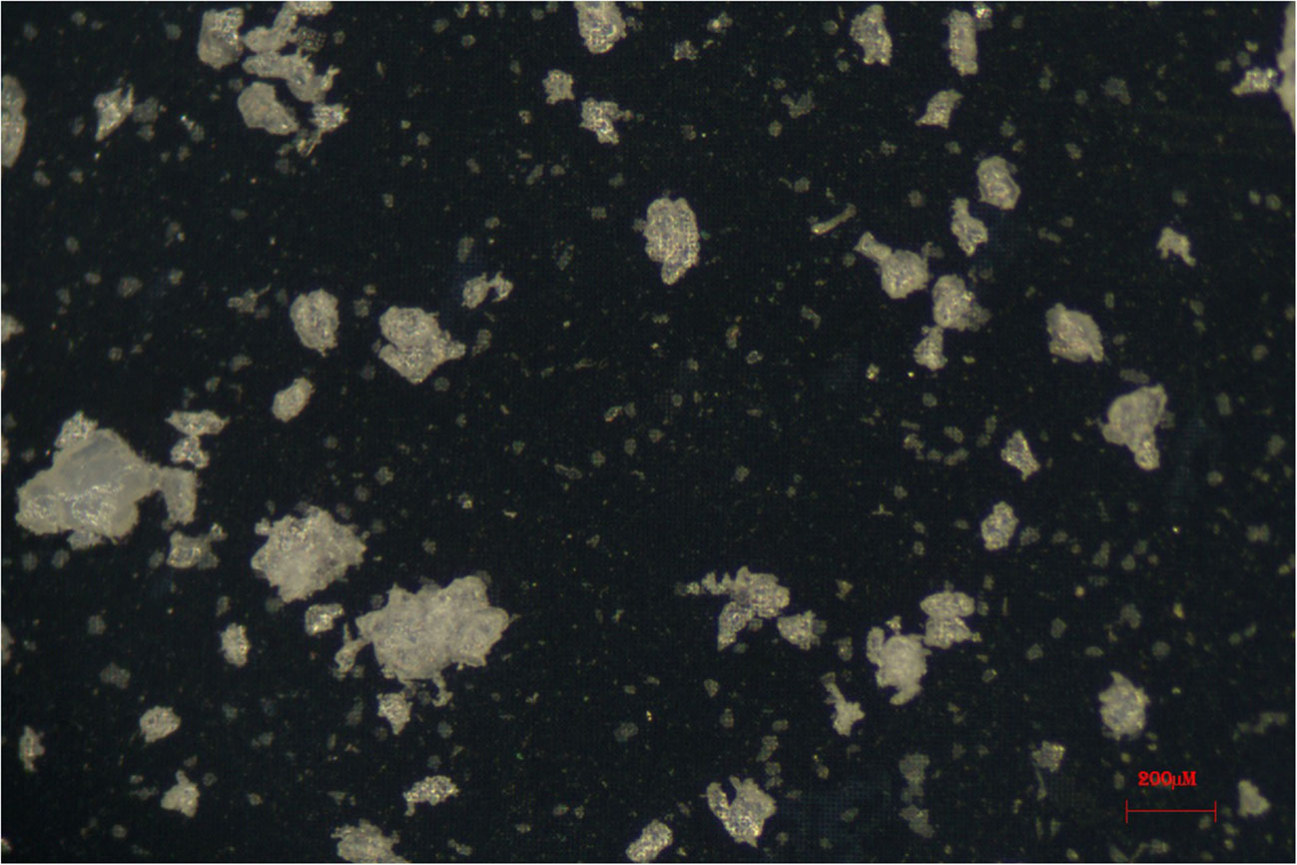
Close-up of PET particles on macroporous silica membrane with × 40 magnification. The 5 μm pores with the 12 μm inter-distance raster is just barely visible

In Figs. 4 and 5, micrographs of the macroporous silica membranes are presented. The images show that the contamination of the water and the surfactant was low but not completely absent. In Fig. 4a, an empty macroporous silica membrane is shown. It is completely free of any visible contamination. In the remaining figures (Fig. 4b to d), the material is mainly observed inside a circle resulting from the two sealing rings used for the vacuum filtration. Any particle present outside the circle had accidentally moved out of it, because of handling and drying of the silica membranes. In Fig. 4b and c, the circle can be discerned with some difficulty indicating relatively clean samples. In Fig. 4b, the silica membrane is shown after 50 mL of the Triton X-100 surfactant solution was passed through. Since all filtering took place in a clean bench, the contamination from fibres is from the preparation and filling step of that solution. Then, Fig. 4c shows a filter after 950 mL of type 2 water was passed through. Five larger particles are apparent, but no fibres were present. The level of contamination is much below 1% of the number of particles that can be seen in Fig. 4d (5 out of 800). In Figs. 4d and 5, irregular PET particles from one NaCl carrier can be observed. Uniform appearance with respect to colour and other features indicates that only PET is present which was the only polymer added to the sample. In fact, Fig. 4d is a combination of two components since 50 mL of Triton X-100 was used to dissolve the NaCl carrier. The magnification in Fig. 5 finally shows a close-up of an area from Fig. 4d. By comparing with the length scale, one can see that the operationally defined selection of particles has rendered relatively uniform particles, although smaller particles of PET are also present. The number of fine particles < 30 μm outstrips the total number of particles in the target range > 30 μm. Even so, the presence of fine particles below 30 μm does not have a significant impact on the total mass of PET as discussed below. As an example, one spherical particle of 2 μm and one of 30 μm diameter differ more than 3300 times in mass. This consideration was part of the design at the outset, i.e. asking the ILC participants only to report easily observable larger particles instead of reporting ‘all’ particles. Figures 4b and 5 show that there is some room for improvement in preventing contamination and reducing the number of fine particles.

### Considerations related to the particle size distributions and mass of PET

**Fig. 6:**
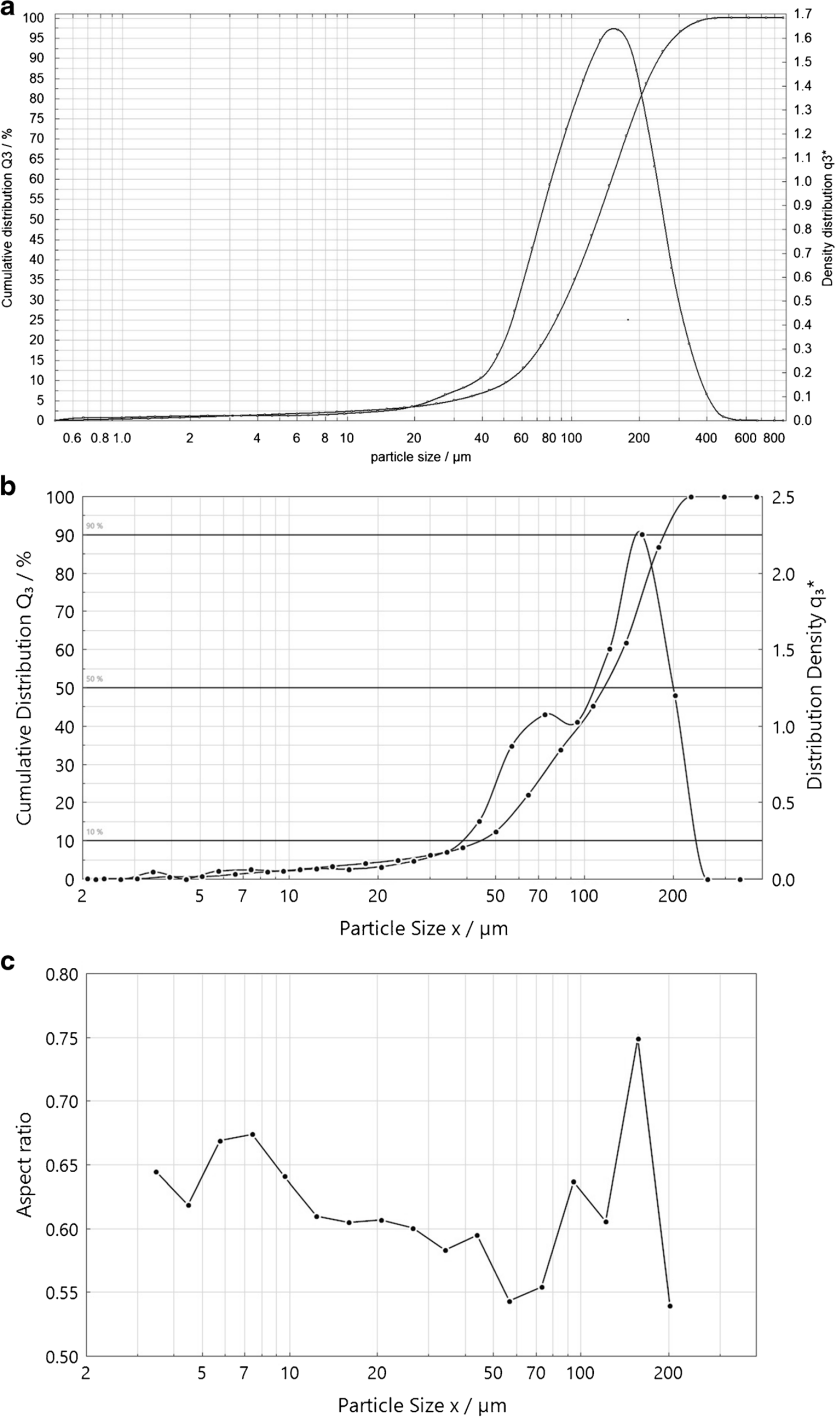
**a** Average particle size distribution obtained using laser diffraction (LD) of 18 combined vials of NaCl carrier with PET (*n* = 3). **b** Average particle size distribution by dynamic image analysis (DIA) measuring 30 combined NaCl carrier samples for 20 s at 20 Hz. **c** Aspect ratio of PET particles as function of particle size obtained by DIA

The particle size distribution measurements shown in Fig. 6a and b and Table S3 reveal that 5.0% and 6.2% of the total volume distribution was < 30 μm using laser diffraction and dynamic image analysis, respectively. (Data for the cumulative distributions using the two different techniques is shown in ESM Table S3.) This means that for the mass recording, about 5% of the mass corresponded to particles outside the interval to be reported for counting. As already mentioned, it was assumed from the beginning that smaller-sized PET particles would be present but at an insignificant level in relative terms to the total mass. To illustrate this, one can consider the error bars in Fig. 3, which are of about the same magnitude as the contribution from the finer particles. Hence, the material was deemed suitable for the ILC that subsequently took place.

When particles are measured by laser diffraction, an equivalent sphere diameter (ESD) is reported. For this reason, the particle volume obtained by laser diffraction is over-estimating the actual volume of all particles since most of the MP particles are non-spherical. This feature is inherent to the technique and it cannot be circumvented. In addition, no information about the particle shape is generated using laser diffraction [23]. An average X_50_ of 131 μm for the cumulative volume distribution was obtained using laser diffraction (*n* = 3). Data was also obtained using dynamic image analysis using the QICPIC instrument to obtain information about the shape of the PET particles. Data from the QICPIC system displayed in Fig. 6b and c show the particle size distribution aspect ratios of the particles ranging from 3.4 to 200 μm. (The aspect ratio is the Feret_min_ diameter divided by the Feret_max_ diameter and is calculated from a 2-dimensional projection onto a plane for all particles.) Particles that are elongated have an aspect ratio < 1. The mean aspect ratio for the data in Fig. 6c is 0.616 which means that the PET particles are elongated as can also be observed in Figs. 4d and 5.

A calculation was made to see if the obtained mass of PET was realistic. Therefore, the average particle volume was multiplied by 800 particles in the specified interval. A mean diameter of 80 μm (average aspect ratio 0.616 × 131 μm = 81 μm) was used instead of directly using the X_50_ of 131 μm to calculate the mean volume. This size of particle is below the mid-range in the particle size distribution of the target interval shown in Fig. 6a and b. The calculated result is 300 μg (ρ for PET = 1.4 g/cm^3^) which is very close to the mean of all weighings of 293 μg. The calculated mass based on data from the particle size distributions and particle shape consequently corresponds well with the mass obtained by weighing.

When applying 300 μg of PET per NaCl carrier sample, it results in a total mass of 0.16 g of PET in the 521 filled vials. Since about half of the volume of the original stock was used, one can assume that about 0.32 g of PET was present in the original litre of stock solution. Considering the original 2 g of PET in suspension, one can conclude that about 60% of the mass had been lost in the subsequent wet-sieving steps. Weighing of the refined (and dried) PET fraction before final resuspension was not attempted as the PET particles are notoriously electrostatic in dry powder form. In the future work, it would be desirable to try to work around this problem to obtain a complete mass budget.

### Confirmation of PET by spectroscopic techniques

The polymer composition of the particulate material in the NaCl carrier samples was identified as PET by FT-IR and Raman spectrometry. This excludes the possibility of any mix-up or mistake during sample preparation. Comparisons with reference PET and a reference spectrum are shown in Fig. S2. Hence, the identity of the measurand was unambiguously confirmed [24].

## Conclusions

We have shown that it is possible to rapidly prepare a reference material for MP particles that was sufficiently homogeneous and stable to be of meaningful use in a large ILC. One major advantage of the approach is that the solid NaCl carrier immobilises the small MP particles during transport and handling. Only just before the sample will be analysed, reconstruction should take place. We think this is a viable approach for preparing many kinds of different standards in the field of MP measurements which up to now is suffering from poor comparability and lack of harmonisation. It is assumed that this is a better approach than filling > 500 bottles with 1 L of water and then add 800 ± 150 MP particles per bottle (or vice versa). We have also presented calculated data based on laser diffraction and dynamic image analysis measurements that underpin other measurement results of number and mass of PET particles. By using this concept, preparation of other reference materials containing different polymers or mixtures of polymers would be possible as well with different number of particles in specific particle size ranges. The potential for upscaling must be verified before even larger batches can be prepared but we have no reason to believe that this will be problematic. Additional developments should also focus on trying to reduce the number of fine particles and to further reduce contamination during preparation.

## Supplementary information

ESM 1 (PDF 636 kb).
