# Supplementary material for: Preparation of a reference material for microplastics in water—evaluation of homogeneity
Source: Anal Bioanal Chem. 2021 Feb 6;414(1):385–97. doi: 10.1007/s00216-021-03198-7 (PMC8748356; doi:10.1007/s00216-021-03198-7)
Supplement: Supplementary file 1 — (PDF 636 kb). [file 216_2021_3198_MOESM1_ESM.pdf]

Table S1. Counting data of PET microplastics > 30 µm in reconstituted[1] water samples or by direct analysis[2] of salt carrier, reported per vial.

Vial number	JRC-Ispra[1,3]	JRC-Geel[2]	External laboratory[1]	Mass of NaCl, g
3	988			0.284
27			875	
37		765		0.294
81			831	
101	784			0.296
119	834			0.292
123		693		0.295
143			714	
164		826		0.295
168	622			0.263
204			819	
243	625			0.285
252			821	
264		778		0.279
265	712			0.281
280			1106	
327		887		0.268
342	629			0.269
348			801	
400	696			0.278
416			683	
436			**1167**	
445	783			0.280
469		792		0.282
507			835	
508	625			0.282
509		816		0.284

[3]Reported result is the mean value of four different measurements of the same filter.

Table S2. Mass of PET microplastics in the NaCl-carrier per vial.

Vial number	Mass of PET, µg	Expanded uncertainty, U, µg
6	296.8	12
15	349.7	12
92	241.5	19
132	319.6	8.9
177	341.8	37[1]
222	260.5	8.4
258	322.6	11
288	340.8	15
359	256.6	36[1]
389	237.1	11
418	277.1	45[1]
434	314.7	9.3
441	303.5	25
466	238.2	10

[1]Increased uncertainty of the weighing due to possible loss of PET. More details about the substitution weighing are given in the experimental section and in results and discussion.

Table S3. Cumulative volume distributions of PET in the NaCl carrier using laser diffraction (LD) and dynamic image analysis (DIA). In both cases the average of three replicate measurements is reported for 15 or 30 samples of resuspended NaCl-carrier. Data in bold is relevant to the particle sizes >30 µm to be reported using counting methods.

Size classes for LD, µm	Cumulative volume distribution using LD[1], %	Size classes for DIA, µm	Cumulative volume distribution using DIA, %
2.2	0.85	2.07	0.16
2.6	0.98	2.36	0.16
3.1	1.13	3.05	0.16
3.7	1.28	3.93	0.68
4.3	1.4	5.07	0.70
5	1.53	6.53	1.29
6	1.68	8.42	1.98
7.5	1.89	10.86	2.57
9	2.08	14.00	3.34
10.5	2.27	18.05	4.06
12.5	2.51	23.28	4.96
15	2.8	**30.02**	**6.25**
18	3.15	**38.70**	**8.21**
21	3.54	**49.90**	**12.40**
25	4.12	**64.34**	**22.02**
**30**	**4.97**	**82.96**	**33.09**
**36**	**6.04**	**106.96**	**45.25**
**43**	**7.4**	**137.92**	**61.86**
**51**	**9.43**	**177.83**	**86.73**
**61**	**13**	**229.28**	**100.00**
**73**	**18.61**	**295.63**	**100.00**
**87**	**26.12**		
**103**	**35.05**		
**123**	**46.01**		
**147**	**58.32**		
**175**	**70.68**		
**215**	**83.75**		
**255**	**91.61**		
**305**	**96.57**		
**365**	**99.05**		
**435**	**99.89**		

[1]More data exists from 0.5 µm upwards, but was omitted to match the measurement range of DIA

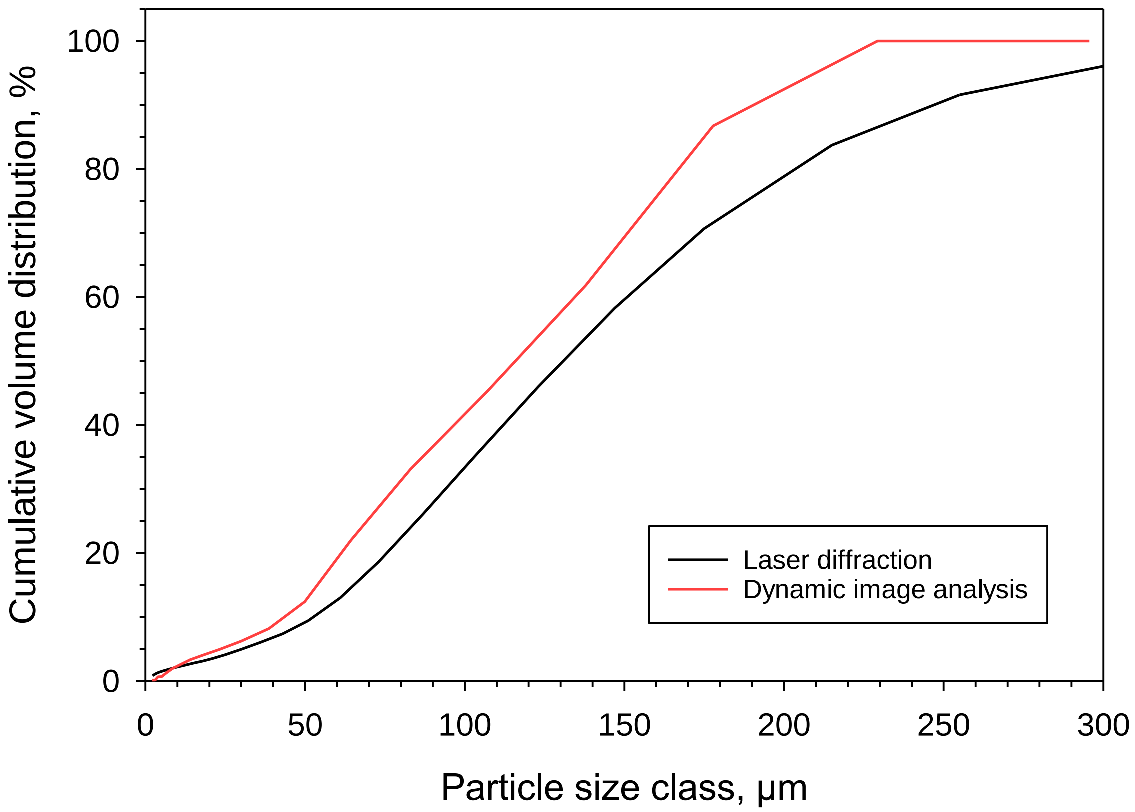

Fig. S1. Comparison of cumulative volume distributions for PET-particles using DIA and LD from 2 µm upwards, (also reported in Table S3).

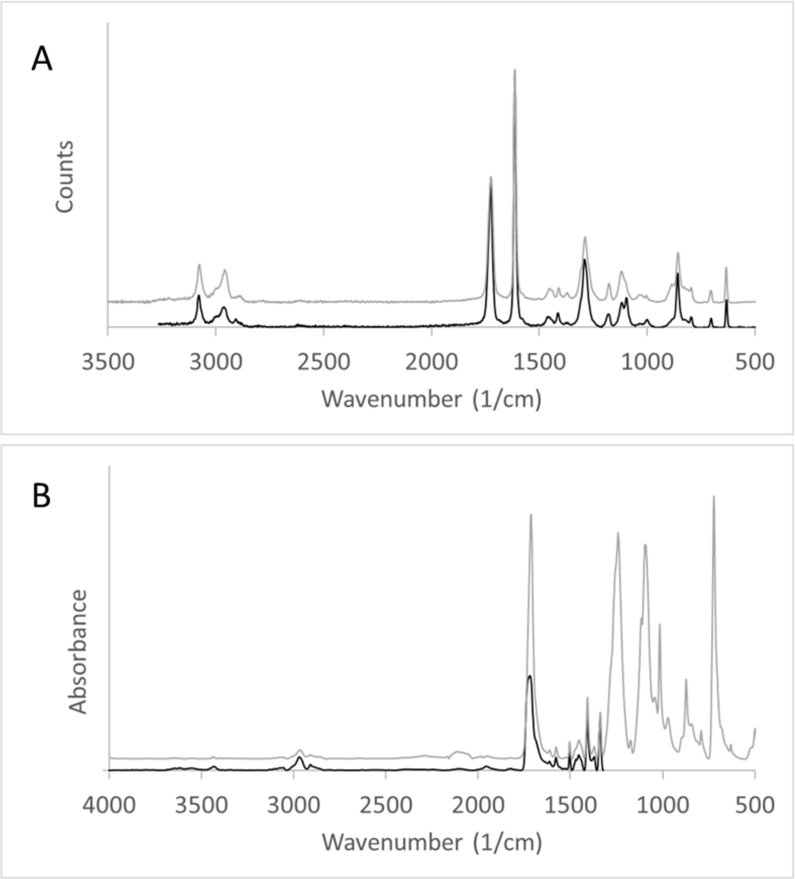

Fig. S2. (A) Raman spectrum of the PET particles (black) and the reference polymer (Goodfellow in grey). (B) FT-IR spectra of PET particles (black) and reference spectrum of PET from spectral library (grey).
